# Improved Object Detection Artificial Intelligence Using the Revised RetinaNet Model for the Automatic Detection of Ulcerations, Vascular Lesions, and Tumors in Wireless Capsule Endoscopy

**DOI:** 10.3390/biomedicines11030942

**Published:** 2023-03-17

**Authors:** Ayako Nakada, Ryota Niikura, Keita Otani, Yusuke Kurose, Yoshito Hayashi, Kazuya Kitamura, Hiroyoshi Nakanishi, Seiji Kawano, Testuya Honda, Kenkei Hasatani, Tetsuya Sumiyoshi, Tsutomu Nishida, Atsuo Yamada, Tomonori Aoki, Tatsuya Harada, Takashi Kawai, Mitsuhiro Fujishiro

**Affiliations:** 1 Department of Gastroenterology, Graduate School of Medicine, The University of Tokyo, Tokyo 1138655, Japan; 2 Department of Gastroenterological Endoscopy, Tokyo Medical University Hospital, Tokyo 1600023, Japan; 3 Research Center for Advanced Science and Technology, The University of Tokyo, Tokyo 1538904, Japan; 4 Department of Gastroenterology and Hepatology, Osaka University Graduate School of Medicine, Osaka 5650871, Japan; 5 Department of Gastroenterology, Kanazawa University Hospital, 9208641 Kanazawa, Japan; 6 Department of Gastroenterology, Ishikawa Prefectural Central Hospital, Ishikawa 9208530, Japan; 7 Department of Gastroenterology and Hepatology, Okayama University Graduate School of Medicine, Dentistry, and Pharmaceutical Sciences, Okayama 7008558, Japan; 8 Department of Gastroenterology, Nagasaki Harbor Medical Center, 8508555 Nagasaki, Japan; 9 Department of Gastroenterology, Fukui Prefectural Hospital, Fukui 9108526, Japan; 10 The Center for Digestive Disease, Tonan Hospital, Sapporo 0600004, Japan; 11 Department of Gastroenterology, Toyonaka Municipal Hospital, 5608565 Toyonaka, Japan

**Keywords:** artificial intelligence, small intestine erosions and ulcers, small intestine tumors, small intestine vascular lesions, wireless capsule endoscopy

## Abstract

The use of computer-aided detection models to diagnose lesions in images from wireless capsule endoscopy (WCE) is a topical endoscopic diagnostic solution. We revised our artificial intelligence (AI) model, RetinaNet, to better diagnose multiple types of lesions, including erosions and ulcers, vascular lesions, and tumors. RetinaNet was trained using the data of 1234 patients, consisting of images of 6476 erosions and ulcers, 1916 vascular lesions, 7127 tumors, and 14,014,149 normal tissues. The mean area under the receiver operating characteristic curve (AUC), sensitivity, and specificity for each lesion were evaluated using five-fold stratified cross-validation. Each cross-validation set consisted of between 6,647,148 and 7,267,813 images from 217 patients. The mean AUC values were 0.997 for erosions and ulcers, 0.998 for vascular lesions, and 0.998 for tumors. The mean sensitivities were 0.919, 0.878, and 0.876, respectively. The mean specificities were 0.936, 0.969, and 0.937, and the mean accuracies were 0.930, 0.962, and 0.924, respectively. We developed a new version of an AI-based diagnostic model for the multiclass identification of small bowel lesions in WCE images to help endoscopists appropriately diagnose small intestine diseases in daily clinical practice.

## 1. Introduction

Wireless capsule endoscopy (WCE) is a revolutionary examination method that can evaluate the entire 6 m long small intestine [1]. The American, European, and Japanese societies for gastrointestinal endoscopy recommend WCE as the primary examination for patients with obscure gastrointestinal bleeding, small intestine tumors, and inflammatory bowel disease. Although a single WCE examination can acquire 10,000–80,000 images, only a few abnormal images are required to diagnose small intestine lesions. The most important issue related to WCE images is low inter- and intra-observer agreement [2]. A recent meta-analysis reported 0.6–0.79 inter-observer agreement in 56% of the WCE examinations for small intestine lesions. The diagnostic yield of WCE depends on the examination time [3] and the endoscopist’s skill and experience [4]. Longer examination times and lack of experience may lead to lower diagnostic yields. Furthermore, there are currently no standardized diagnostic protocols or reporting systems. Thus, new diagnostic solutions are required to improve the accuracy of WCE diagnoses.

Artificial intelligence (AI) models can be used to improve the diagnostic accuracy of diseases of the small intestine [5,6]. We previously reported on the high diagnostic accuracy of an AI model that we developed, RetinaNet, for identifying small intestine erosions and ulcers, angioectasias, and tumors [5]. Although the model has high diagnostic yield, it may at times indicate false positive/negative results from the images. Generally, improvements in the diagnostic accuracy of AI models require an increase in the data size. Therefore, we developed a new RetinaNet model using the largest dataset in the world, consisting of >10,000,000 WCE images obtained from nine hospitals.

## 2. Materials and Methods

### 2.1. Study Sample and Preparation of the Image Set

We performed a retrospective study using a WCE database. First, we collected WCE images acquired between April 2009 and July 2019 from the University of Tokyo Hospital from patients with obscure gastrointestinal bleeding, possible small intestine tumors, or abdominal symptoms. We previously used these data to develop the RetinaNet model [5]. We expanded the angioectasia WCE database by adding images acquired between 2009 and 2019 at Ishikawa Prefectural Central Hospital, Fukui Prefectural Hospital, Tonan Hospital, the University of Okayama Hospital, the University of Kanazawa Hospital, Nagasaki Medical Center, the University of Osaka Hospital, and Toyonaka Municipal Hospital for obscure gastrointestinal bleeding, examination for small intestine tumors, or abdominal symptoms (Table 1). All WCE procedures used the PillCam SB2 or SB3 capsule endoscope (Medtronic, Minneapolis, MN, USA) and were carried out after patients had fasted for 12 h. Oral simethicone (40 mg) was administered before the WCE examinations [7].

From the database, we extracted a case group of 651 patients with erosions and ulcers, angioectasias, or tumors. We also randomly extracted a control group of 482 patients and normal images from these patients.

**Figure 1:**
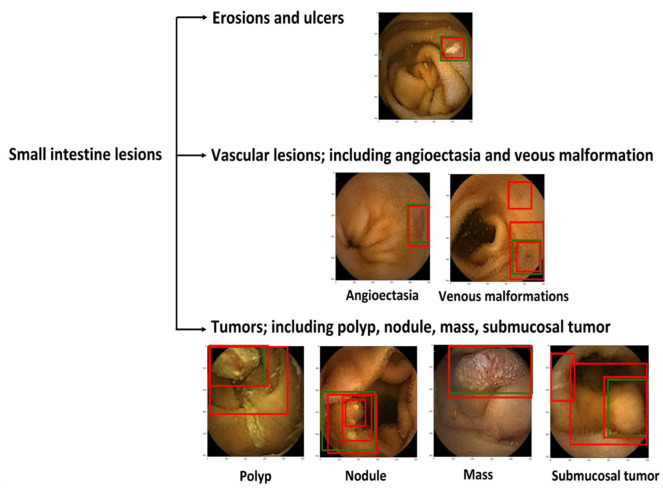
Classification of small intestine lesions. Green boxes, gold-standard bounding boxes; red boxes, AI-detected bounding boxes.

The WCE images were used to develop a dataset, consisting of 6476 images of erosions and ulcers, 1916 of angioectasias, 7127 of tumors, and 14,014,149 normal images. This study was approved by all of the participating hospitals (No. 12016-1). A vascular lesion was defined as angioectasias and venous malformations; a tumor was defined as a polyp, nodule, mass, and/or submucosal tumor (Figure 1). Four expert WCE endoscopists (AN, RN, TA, and AY) manually annotated all lesions with bounding boxes (gold-standard boxes). All annotations were performed independently, and any disagreement was resolved by consensus.

### 2.2. RetinaNet Algorithm

**Figure 2:**
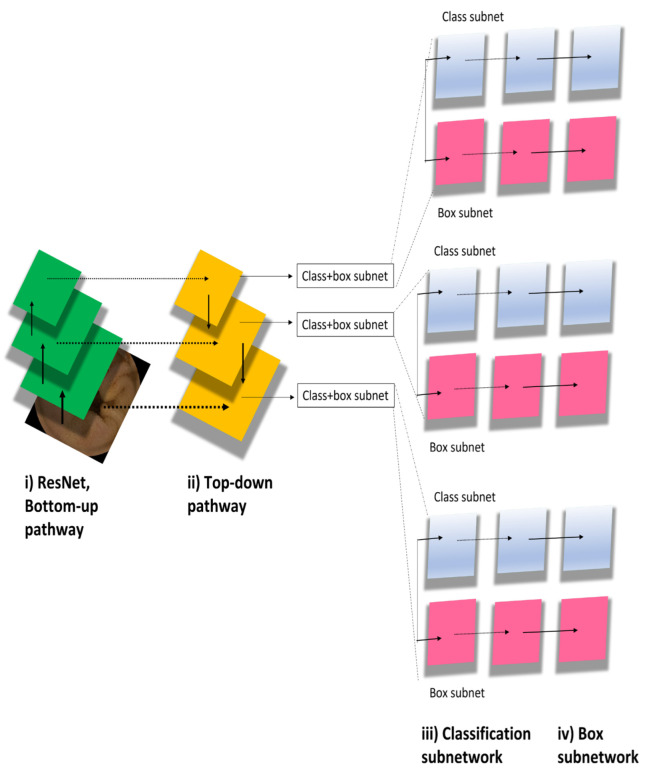
RetinaNet model algorithm.

We used the deep neural network architectures of RetinaNet [8] to develop a new AI-based diagnostic model. The major RetinaNet network included ResNet, bottom-up pathway, top-down pathway, classification subnetwork, and box subnetwork (Figure 2). The RetinaNet network architecture uses a Feature Pyramid Network backbone on top of a feedforward ResNet architecture to construct a rich, multiscale convolutional feature pyramid. RetinaNet attaches two subnetworks: one for classifying anchor boxes and another for regressing from anchor boxes to ground-truth object boxes. We trained the RetinaNet model to detect areas within the bounding boxes as lesions and those outside of the boxes as background. The input image size was 512 × 512. Learning was carried out by penalizing incorrect outputs and iteratively minimizing this penalty. Notably, lesion detection differs from general object detection in that the boundaries of the detection targets are ambiguous. The penalty was relaxed to allow some positional shifting of the output boxes.

Previously, we had developed the RetinaNet model using the data of 398 erosion and ulceration images, 538 angioectasias images, 4590 tumor images, and 34,437 normal images from a single hospital [5]. In the current study, we further trained the model using 6476 erosion and ulcer images, 1916 angioectasias images, 7127 tumor images, and 14,014,149 normal images from nine hospitals.

### 2.3. Outcome Measures and Statistics

**Figure 3:**
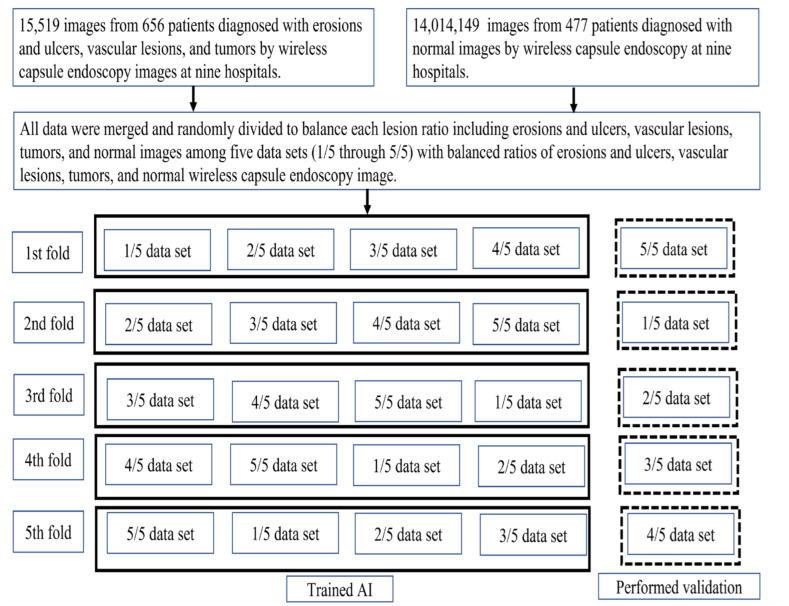
Study flow diagram.

The primary outcome was a per-lesion image diagnosis of small intestine lesions including erosions and ulcers, vascular lesions, and tumors. The model accuracy was defined based on the overlap between the AI-drawn bounding boxes and the gold-standard boxes. We used five-fold stratified cross-validation to balance the lesion ratios to test the model (Figure 3). When generating the internal and external validation sets, random sampling was performed to avoid bias that could lead to false readings regarding the model’s performance. The trained RetinaNet model drew red bounding boxes (AI boxes) around lesions detected in the validation set, and output probability scores ranged from 0 to 1 for each erosion, ulceration, vascular lesion, and tumor; the higher the score, the greater the confidence that the region included a lesion of the specified type. The following definitions were used to assess model accuracy. First, any overlap between the AI box and the gold-standard box was considered positive. Second, if several AI boxes were created in a single image and even one of them detected a lesion, image classification was considered accurate.

We plotted receiver operating characteristic (ROC) curves and estimated areas under the ROC curve (AUC) and 95% confidence intervals (CIs), sensitivity, specificity, and the accuracy of the AI detection model for each lesion image for each probability score cutoff of the Youden index. The mean AUC, sensitivity, and specificity were estimated using the fold data.

The secondary outcomes were the per-intersection over union (IOU) and per-patient diagnosis of the three lesional types. During per-lesion IOU analyses, we defined the area of overlap divided by the area of union as the IOU. We calculated the IOUs for all lesions in each cross-validation set, and then estimated the mean IOU for each lesion. During per-patient analyses, we estimated the number of affected patients and the rates of AI-detected lesions in each cross-validation set. All statistical analyses were performed with Python (ver. 3).

## 3. Results

### 3.1. Per-Lesion Image Analyses

**Figure 4:**
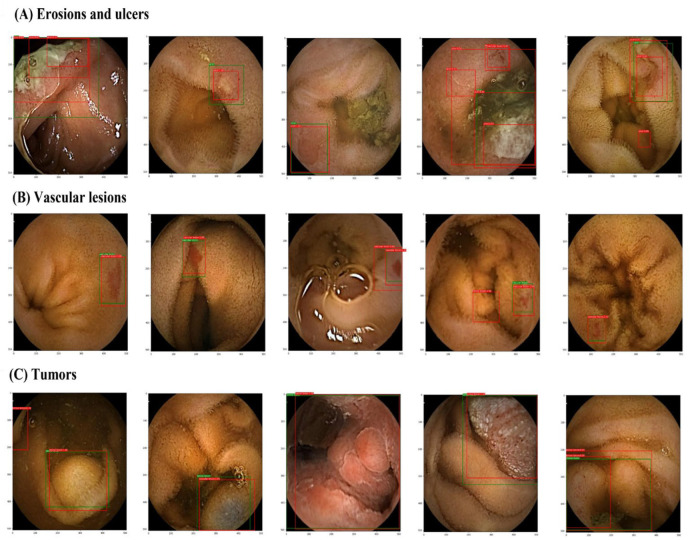
Images of small intestine erosions and ulcers, vascular lesions, and tumors diagnosed by artificial intelligence (AI). (**A**) erosions and ulcers, (**B**) vascular lesions, and (**C**) tumors. Green boxes, gold-standard bounding boxes; red boxes, AI-detected bounding boxes.

**Figure 5:**
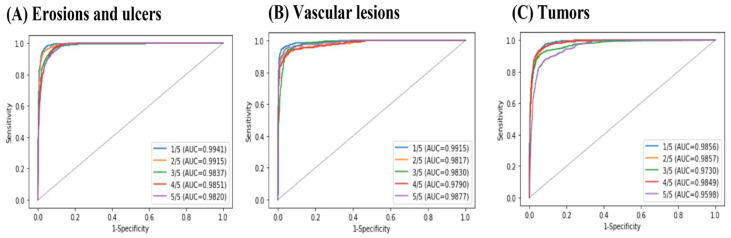
Receiver operating characteristic (ROC) curves and area under the ROC curve (AUC) values for small intestine lesions: (**A**) erosions and ulcers, (**B**) vascular lesions, and (**C**) tumors.

The number of patients from each of the nine institutions is shown in Table 1. Each cross-validation set consisted of between 6,647,148 and 7,267,813 images from 217 patients. The lesion and normal image ratios were well balanced among the cross-validation sets. Images of small intestine erosions and ulcers, vascular lesions, and tumors diagnosed by artificial intelligence (AI) are shown in Figure 4. The mean AUC values were 0.997 for erosions and ulcers, 0.998 for vascular lesions, and 0.998 for tumors (Figure 5). The mean sensitivity values were 0.919, 0.878, and 0.876; the mean specificities were 0.936, 0.969, and 0.937; and the mean accuracies were 0.930, 0.962, and 0.924, respectively (Table 2).

### 3.2. Per-Lesion IOU Analyses

The mean IOU of RetinaNet was 0.839 (95% CI = 0.792, 0.886) for erosions and ulcerations, 0.833 (95% CI = 0.780, 0.886) for vascular lesions, and 0.798 (95% CI = 0.750, 0.846) for tumors. The IOU values for each type of lesion in each cross-validation set are shown in Table 3.

### 3.3. Per-Patient Analyses

The per-patient diagnoses in each cross-validation fold are shown in Table 4. The AI model missed three, three, three, four, and one patient for erosions and ulcers in the first to fifth cross-validation folds, respectively; one patient for vascular lesions in the fourth fold; and one, one, and two patients for tumors in the third, fourth, and fifth folds, respectively.

## 4. Discussion

We improved our AI model RetinaNet to detect all types of small-bowel lesions in WCE images. We further trained the model using a larger number of WCE images obtained from nine institutions. Currently, the model shows the highest performance for diagnostic yield for WCE examination among object-detection AI models [9].

### 4.1. Improved Specificity and Accuracy of Tumor Detection

Our current RetinaNet model is better than the original in terms of tumor detection. For the previous RetinaNet model, the mean specificity was 0.918 (95% CI = 0.881–0.955) and the mean accuracy was 0.914 (95% CI = 0.879–0.950) [5]. For the current model, the mean specificity was 0.937 (95% CI = 0.926–0.948) and the mean accuracy was 0.924 (95% CI = 0.911–0.936). Small intestine tumors are rare. The increased number of WCE images from the nine hospitals improved the diagnostic yield.

### 4.2. High Specificity of the RetinaNet Algorithm for All Types of Lesion

Our RetinaNet model has considerable strength with high specificity, given the extended AI training on each lesion type. It learns the features of normal images using weakly supervised learning; this allows for improved accuracy, as the total number of training images can be easily accommodated. In the current study, more than 10,000,000 normal images were used for training, as a previous meta-analysis reported an association between higher specificity and a larger number of training images in WCE AI models [9]; moreover, AI models trained using a total number of training images >20,000 show the lowest false-positive rate in small WCE examinations [9].

### 4.3. Future Tasks

Further validation analyses are required to evaluate various AI models for WCE image assessment. Such analysis should ideally use open-source codes and directly compare AI models using the same dataset. Comparisons using publicly available common datasets or meta-analyses may also be effective.

We have also planned to use the current version of the RetinaNet model in a clinical setting. The model can diagnose WCE images, but not videos. Therefore, we have developed an original, comprehensive, user-interface network system, including video-to-image conversion, that shows abnormal images classified by the lesion type and identifies their location in the small intestine. Endoscopists can obtain RetinaNet-detected results using the web-based system anytime and anywhere. We plan to use this system in hospitals that may be willing to participate in the research.

### 4.4. Limitations

First, this study used a retrospective design. Next, the diagnostic yield of our former RetinaNet model showed good performance, but had reached a plateau level regarding learning effects. Specifically, the improvement in diagnostic yield, in terms of sensitivity, specificity, and accuracy for erosions, ulcers, and vascular lesions, was limited, although the number of training images increased by twenty-, four-, and two-fold, respectively. Furthermore, it missed several patients with erosions and ulcers, one patient with a vascular lesion, and one patient with a tumor. These missed features would be difficult to diagnose, even for expert endoscopists. The diagnostic yield of small intestine lesions using the current AI model does not reach that of expert endoscopists; however, in current clinical practice, we believe that it would be effective as a first screening tool for endoscopists reading WCE videos or as a means of cross-checking image findings after an endoscopist’s reading of WCE videos.

## 5. Conclusions

We developed a new version of the RetinaNet model for the multiclass diagnosis of lesions in WCE images. The improved version of our model will be especially useful for endoscopists to appropriately diagnose small intestine disease in daily clinical practice.

## Figures and Tables

**Table 1:** Number of patients from each of the nine institutions.

Hospital	Erosions and Ulcers	Vascular Lesions	Tumors	Normal
University of Tokyo Hospital	161	19	73	314
Ishikawa Prefectural Central Hospital	51	21	20	142
Fukui Prefectural Hospital	6	0	3	0
Tonan Hospital	2	2	0	2
Osaka University Hospital	22	3	16	0
University of Kanazawa Hospital	127	32	26	6
Nagasaki Minato Hospital	11	6	4	13
University of Osaka Hospital	96	28	26	0
Toyonaka Hospital	1	0	1	0
Total	477	111	169	477

**Table 2:** Per-image analysis of mean sensitivity, specificity, and accuracy.

	Sensitivity	Specificity	Accuracy
Erosions and ulcers	0.919 (0.896–0.942)	0.936 (0.914–0.957)	0.930 (0.912–0.953)
Vascular lesions	0.878 (0.823–0.933)	0.969 (0.958–0.979)	0.962 (0.951–0.973)
Tumors	0.876 (0.840–0.912)	0.937 (0.926–0.948)	0.924 (0.911–0.936)

Parentheses contain 95% confidence intervals.

**Table 3:** IOU values for each type of lesion in each cross-validation set.

	First Fold	Second Fold	Third Fold	Fourth Fold	Fifth Fold
Erosions and ulcers	0.8893	0.8972	0.7792	0.7959	0.8354
Vascular lesions	0.9155	0.8490	0.7913	0.7604	0.8511
Tumors	0.7991	0.8132	0.8455	0.8297	0.7061

**Table 4:** Number of diagnosed small intestine lesions according to the number of patients analyzed.

	First Fold	Second Fold	Third Fold	Fourth Fold	Fifth Fold
Erosions and ulcers	64/67	65/68	65/68	63/67	73/74
Vascular lesions	27/27	30/30	25/25	34/35	29/29
Tumors	36/36	29/29	36/37	27/28	26/28

## Data Availability

The data presented in this study are available on reasonable request to the corresponding author.
